# Rigid Macrocycle Metal Complexes as CXCR4 Chemokine Receptor Antagonists: Influence of Ring Size

**DOI:** 10.3390/pharmaceutics16081000

**Published:** 2024-07-28

**Authors:** Isaline Renard, Thomas D’huys, Benjamin P. Burke, Trisha Ajoleza, Amy N. Cain, Neil L. Funwie, Abid Khan, Danny L. Maples, Randall D. Maples, Dallas L. Matz, Graeme McRobbie, Robert Ullom, Timothy J. Prior, Douglas P. Linder, Tom Van Loy, Timothy J. Hubin, Dominique Schols, Stephen J. Archibald

**Affiliations:** 1 Centre for Biomedicine and Positron Emission Tomography Research Centre, The University of Hull, Cottingham Road, Hull HU6 7RX, UK; 2 School of Biomedical Engineering and Imaging Sciences, King’s College London, 4th Floor Lambeth Wing, St Thomas’ Hospital, London SE1 7EH, UK; 3 Division of Virology and Chemotherapy, Rega Institute for Medical Research, KU Leuven, B-3000 Leuven, Belgium; 4 Department of Chemistry and Physics, Southwestern Oklahoma State University, Weatherford, OK 73096, USA; 5 Division of Pharmacy and Optometry, School of Health Sciences, Faculty of Biology, Medicine and Health, The University of Manchester, Manchester M13 9PL, UK; 6 Chemistry, Faculty of Science and Engineering, The University of Hull, Cottingham Road, Hull HU6 7RX, UK

**Keywords:** CXCR4, AMD3100, Plerixafor, cyclam, macrocycle, cancer therapeutics, molecular imaging

## Abstract

Understanding the role of chemokine receptors in health and disease has been of increasing interest in recent years. Chemokine receptor CXCR4 has been extensively studied because of its defined role in immune cell trafficking, HIV infection, inflammatory diseases, and cancer progression. We have developed high affinity rigidified CXCR4 antagonists that incorporate metal ions to optimize the binding interactions with the aspartate side chains at the extracellular surface of the CXCR4 chemokine receptor and increase the residence time. Cross- and side-bridged tetraazamacrocylic complexes offer significant advantages over the non-bridged molecular structures in terms of receptor affinity, potential for radiolabelling, and use in therapeutic applications. Our investigation has been extended to the influence of the ring size on bridged tetraazamacrocyclic compounds with the addition of two novel chelators (bis-cross-bridged homocyclen and bis-cross-bridged cyclen) to compare to the bis-bridged cyclam, along with novel metal complexes formed with copper(II) or zinc(II). The in vitro biological assays showed that all of the zinc(II) complexes are high affinity antagonists with a marked increase in CXCR4 selectivity for the bis-cross-bridged cyclen complex, whereas the properties of the copper(II) complexes are highly dependent on metal ion geometry. X-ray crystal structural data and DFT computational studies allow for the rationalisation of the relative affinities and the aspartate residue interactions on the protein surface. Changing the ring size from 14-membered can increase the selectivity for the CXCR4 receptor whilst retaining potent inhibitory activity, improving the key pharmacological characteristics.

## 1. Introduction

Chemokine receptors, belonging to the family of G protein-coupled receptors (GPCRs), are involved in a wide variety of diseases including cancer and HIV/AIDS as well as inflammatory disorders such as multiple sclerosis, rheumatoid arthritis, and inflammatory bowel disease [1,2,3,4,5]. Their endogenous ligands, chemokines, are small, water-soluble proteins (8–12 kDa) that bind to their cognate receptor and thereby elicit a cellular response. In general, chemokines and chemokine receptors are promiscuous in their interactions. However, chemokine receptor CXCR4 has a sole natural ligand, CXCL12, and, in cancer, this interaction can facilitate metastasis to secondary sites, promote tumour growth, and therapy resistance [6,7,8,9]. CXCR4 has been reported to be overexpressed in more than 23 different human cancers including non-small cell lung, breast, ovarian, prostate, pancreatic, and colorectal cancer [7,10,11,12,13,14,15].

Multiple SAR studies have been carried out to produce selective small molecule chemokine receptor antagonists [16]. Research has also indicated that cancer progression can be mediated by such small molecule receptor antagonists [9,17]. Furthermore, the expression level of CXCR4 has been linked to cancer progression and resistance to radio-/chemotherapy, indicating that it may also be a valuable prognostic biomarker for use in the molecular imaging of cancer growth, metastasis, and relapse [9,14,18,19,20]. Radiolabelled CXCR4 binding ligands have recently been developed for targeted radionuclide imaging and radioisotope therapy [21,22,23].

**Figure 1:**
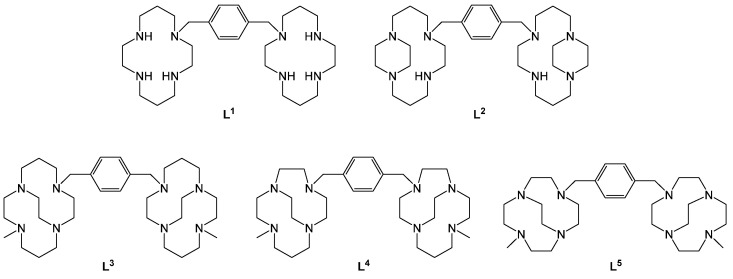
Chemical structures of ligands described in this work.

It has previously been shown that saturated tetraazamacrocyclic structures bind to CXCR4 with a remarkably high affinity. AMD3100 (Plerixafor™, **L^1^** in Figure 1) was initially discovered as a highly specific CXCR4 inhibitor for blocking the HIV cell entry process and was eventually licensed for clinical use in 2008 as a hematopoietic stem cell mobilising agent in patients with multiple myeloma or lymphoma [24]. The high affinity binding interaction of azamacrocyclic antagonists, which are protonated at physiological pH, is achieved via multiple H-bonds and electrostatic interactions with amino acid residues on the accessible extracellular surface of the CXCR4 receptor [25]. Site-directed mutagenesis studies have shown that bis-azamacrocyclic compounds have particularly strong interactions with Asp^171^ in transmembrane loop IV and Asp^262^ in transmembrane loop VI. H-bonding interactions with Glu^288^ and Tyr^45^ as well as π-interactions with Trp^94^ and Tyr^116^ have also been identified [26,27,28].

Macrocyclic chelators such as the 14-membered cyclam ring in AMD3100 can form stable complexes with transition metal ions, which are a good size match with the chelator cavity size. It is proposed that these metal complexes interact with the carboxylate amino acid side chains on the surface of CXCR4 via coordination interactions [29]. The different electronic arrangements of the metal ion result in a range of preferred coordination numbers and optimised geometries. For first row transition metal complexes of cyclam, a flat configuration with all four nitrogens in the same plane and the six-membered chelate rings occupying the chair conformation is most commonly observed, however, it has been proposed that a folded configuration is optimal for binding to the CXCR4 chemokine receptor [30,31,32].

Configurational fixing has been identified as a mechanism to strengthen coordinate bonds with carboxylate functional groups present on the extracellular surface of CXCR4. We used this approach to optimize the interactions of the metal complexes of macrocyclic chelators with CXCR4. This was achieved by adding ethylene bridges between adjacent (side-bridge) or non-adjacent (cross-bridge) nitrogen atoms in the cyclam rings such as **L^2^** and **L^3^**, respectively [33,34,35,36,37]. Furthermore, we demonstrated that there is a significant increase in both potency and residence time at the binding site on the receptor’s extracellular surface [34,38].

In the present study, we expanded the library of configurationally restricted bis-tetraazamacrocylic CXCR4 antagonists and studied their zinc(II) and copper(II) complexes to probe the effect of ring size on the receptor binding characteristics including affinity and selectivity [39,40]. This study will facilitate robust compound selection of a lead drug candidate for translation to in vivo studies, based on the understanding of the coordination interactions to rationalize the results of the in vitro biological assays.

## 2. Materials and Methods

1,4,7,10-Tetraazacyclododecane (cyclen) and 1,4,7,10-tetraazatridecane (homocyclen) were purchased from CheMatech (France). All other reagents were purchased from Sigma Aldrich (USA). Elemental analyses were performed by Quantitative Technologies Inc. Electrospray mass spectra were collected on a Shimadzu LCMS 2020 electrospray mass spectrometer. NMR spectra were obtained on a Varian Bruker AVANCE II 300 MHz NMR spectrometer. Single crystal X-ray diffraction data were collected using an imaging plate diffractometer operating with Mo radiation. Routine data collection and processing procedures were adopted. Several compounds used in this study were synthesised using the following procedures in the literature: **L^1^** [41], Cu**L^1^** and Zn**L^2^** [42], **L^2^** and Cu**L^2^** [33], Zn**L^2^** [43], **L^3^** and Cu**L^3^** [34].

### 2.1. Synthesis of Ligands **L^1^**–**L^5^**

Ligands **L^1^**, **L^2^**, and **L^3^** were synthesised using the following procedures in the literature: **L^1^** [41], **L^2^** [33], and **L^3^** [34]. Synthesis of homocyclen-glyoxal (**1**) and cyclen-glyoxal (**2**) was carried out as previously reported by Weisman et al. [44]. Synthesis of *p*-linked cyclen-glyoxal (**4**) was carried out as previously reported by Le Baccon et al. [45].

#### 2.1.1. Synthesis of p-Linked Homocyclen-Glyoxal (**3**)

Homocyclen-glyoxal (**1**) (5.120 g, 0.025 mol) was dissolved in anhydrous acetonitrile (50 mL), then α,α’-dibromo-*p*-xylene (2.998 g, 0.012 mol) was added and stirred for 24 h. The pale-yellow solid was filtered off and rinsed with acetonitrile, then diethyl ether before drying under vacuum. Yield: 7.406 g (88%). ^13^C [^1^H] NMR (75 MHz, D_2_O): δ 17.65 (N-β-CH_2_), 42.00 (N-α-CH_2_), 45.93 (N-α-CH_2_), 46.40 (N-α-CH_2_), 48.03 (N-α-CH_2_), 50.09 (N-α-CH_2_), 52.19 (N-α-CH_2_), 53.83 (N-α-CH_2_), 58.85 (N-α-CH_2_), 59.60 (N-α-CH_2_), 69.68 (C_aminal_), 81.58 (C_aminal_), 128.63 (C_aromatic_), 132.58 (C_aromatic_). Elemental analysis: calculated (%) for C_30_H_48_N_8_Br_2_•2H_2_O: C, 50.28; H, 7.31; N, 15.64. Found: C, 49.93; H, 7.34; N, 15.34. HRMS (ESI): calculated for [C_30_H_48_N_8_Br]^+^, 599.3180; found 599.3162.

#### 2.1.2. Synthesis of **5**

*p*-Xylene linked glyoxal-bridged homocyclen (**3**) was added to anhydrous acetonitrile (500 mL) with stirring. Iodomethane (162 g, 1.1 mol) was added to the suspension in one portion and the mixture was stirred for 5 days at room temperature under nitrogen. A second portion of iodomethane (0.83 g, 5.8 mmol) was added to the mixture, and then the mixture was stirred at room temperature for a further 5 days. At reaction completion, excess iodomethane was removed by bubbling nitrogen through the suspension for 30 min. The yellow solid was filtered off, washed with diethyl ether (5 × 20 mL), and dried in vacuo to obtain the methylated intermediate (**5**) as an orange solid (8.7 g, 74%). ^13^C[^1^H] NMR (75 MHz, D_2_O): δ 18.90 (N-β-CH_2_), 42.16 (N-α-CH_2_), 45.35 (N-α-CH_2_), 46.46 (N-α-CH_2_), 48.25 (N-α-CH_3_), 50.49 (N-α-CH_2_), 51.57 (N-α-CH_2_), 52.33 (N-α-CH_2_), 59.57 (N-α-CH_2_), 60.41 (N-α-CH_2_), 65.16 (N-α-CH_2_), 76.47 (C_aminal_), 78.40 (C_aminal_), 129.00 (C_aromatic_), 133.58 (C_aromatic_). Elemental analysis: calculated (%) for C_32_H_54_N_8_I_4_•0.5C_4_H_10_O•2H_2_O: C, 36.09; H, 5.61; N, 9.90. Found: C, 35.93; H, 5.31; N, 9.74.

#### 2.1.3. Synthesis of **6**

*p*-Xylene linked glyoxal-bridged cyclen (**4**) was added to anhydrous acetonitrile (300 mL) with stirring. Iodomethane (5 g, 7.7 mmol) was added to the suspension in one portion, and then the mixture was stirred for 14 days at room temperature under nitrogen. At the end of the reaction, excess iodomethane was removed by bubbling nitrogen through the suspension for 30 min. The yellow solid was filtered off, washed with diethyl ether (5 × 20 mL), and dried in vacuo to obtain the methylated intermediate (**6**) as a white solid (7.06 g, 89%). ^13^C[^1^H] NMR (75 MHz, D_2_O): δ 41.77 (N-α-CH_2_), 42.06 (N-α-CH_2_), 45.17 (N-α-CH_2_), 45.46 (N-α-CH_2_), 45.74 (N-α-CH_2_), 53.97 (N-α-CH_3_), 58.17 (N-α-CH_2_), 59.27 (N-α-CH_2_), 60.30 (N-α-CH_2_), 64.04 (N-α-CH_2_), 77.03 (C_aminal_), 77.28 (C_aminal_), 128.37 (C_aromatic_), 132.80(C_aromatic_). Elemental analysis: calculated (%) for C_30_H_50_N_8_I_3_Br: C, 36.64; H, 5.12; N, 11.39. Found: C, 36.30; H, 4.93; N, 11.16. MS (ESI): *m*/*z* = 775 [M-2I]^+^; *m*/*z* = 647 [M-3I]^+^.

#### 2.1.4. Synthesis of **L^4^**

The methylated intermediate (**5**) (8.7 g, 8.2 mmol) was dissolved in ethanol (300 mL), and sodium borohydride (18.7 g, 0.49 mol) was slowly added to the suspension as a solid. The clear solution was stirred at room temperature for 14 days and concentrated in vacuo. Water (200 mL) was added, the pH was adjusted to 14 (KOH pellets), and the basic solution was extracted with benzene (5 × 200 mL). The combined organic fractions were dried over MgSO_4_, filtered, and evaporated in vacuo to yield **L^4^** as a yellow oil (3.82 g, 84%). ^13^C[^1^H] NMR (75 MHz, D_2_O): δ 26.54 (N-β-CH_2_), 43.53 (N-α-CH_3_), 52.24 (N-α-CH_2_), 52.69 (N-α-CH_2_), 53.74 (N-α-CH_2_), 54.61 (N-α-CH_2_), 56.02 (N-α-CH_2_), 56.14 (N-α-CH_2_), 56.40 (N-α-CH_2_), 57.23 (N-α-CH_2_, broad; 2 unresolved peaks), 58.27 (N-α-CH_2_), 60.44 (N-α-CH_2_), 128.61 (C_aromatic_), 138.82 (C_aromatic_). Elemental analysis: calculated (%) for C_32_H_58_N_8_•0.67C_6_H_6_•1.67H_2_O: C, 67.88; H, 10.34; N, 17.59. Found: C, 67.70; H, 9.95; N, 17.30. MS (ESI): *m*/*z* = 555 [M]^+^.

#### 2.1.5. Synthesis of **L^5^**

The methylated intermediate (**6**) (7 g, 6.8 mmol) was dissolved in ethanol (300 mL), and sodium borohydride (7.7 g, 0.2 mol) was slowly added to the suspension as a solid. The clear solution was stirred at room temperature for 5 days and concentrated in vacuo. Water (200 mL) was added, the pH was adjusted to 14 (KOH pellets), and the basic solution was extracted with benzene (5 × 200 mL). The combined organic fractions were dried over MgSO_4_, filtered, and evaporated in vacuo to yield **L^5^** as a yellow oil (2.86 g, 80%). ^13^C[^1^H] NMR (75 MHz, D_2_O): δ 42.84 (N-α-CH_3_), 55.60 (N-α-CH_2_), 56.00 (N-α-CH_2_), 56.58 (N-α-CH_2_), 57.38 (N-α-CH_2_), 59.12 (N-α-CH_2_), 60.10 (N-α-CH_2_), 127.81 (C_aromatic_), 138.09 (C_aromatic_). MS (ESI): *m*/*z* = 527 [M+H]^+^.

#### 2.1.6. Preparation of HCl Salts of **L^4^** and **L^5^**

The ligand HCl salt was made by bubbling HCl gas through an ethanol solution, filtering the yellow solid that formed, and washing with ethanol and ether before drying under vacuum. Elemental analysis (**L^4^**): calculated (%) for C_32_H_58_N_8_•7HCl•6H_2_O: C, 41.86; H, 8.45; N, 12.20. Found: C, 42.16; H, 8.56; N, 12.07. Elemental analysis (**L^5^**): calculated (%) for C_30_H_54_N_8_•7HCl•5H_2_O: C, 41.32; H, 8.21; N, 12.85. Found: C, 41.42; H, 8.36; N, 12.58.

### 2.2. Synthesis of Cu(II) and Zn(II) Complexes of **L^1^**–**L^5^**

Metal complexes of **L^1^**, **L^2^**, and **L^3^** were synthesised using the following procedures in the literature: Cu**L^1^** and Zn**L^1^** [42], Cu**L^2^** [33], Zn**L^2^** [43], and Cu**L^3^** [34].

#### 2.2.1. Synthesis of Zn_2_**L^3^**

In an inert atmosphere glove box, **L^3^** (0.583 g, 1 mmol) and anhydrous zinc(II) acetate (0.367 g, 2 mmol) were dissolved in methanol (20 mL) and stirred overnight. The metal complex solution was removed from the glovebox. NH_4_PF_6_ (5 equiv.) was dissolved in methanol (5 mL), filtered, and added to the zinc complex. The mixture was stored in a freezer overnight to facilitate precipitation. The white precipitate was then collected on a glass frit by filtration, washed with methanol then diethyl ether, and dried under vacuum, yielding Zn_2_**L^3^** (0.961 g, 93%). Elemental analysis: calculated (%) for [C_34_H_58_N_8_Zn_2_](CH_3_COO)_2_(PF_6_)_2_•0.7NH_4_PF_6_: C, 36.93; H, 5.77; N, 9.86. Found: C, 36.77; H, 5.98; N, 9.93. MS (ESI): *m*/*z* = 416 [Zn_2_**L^3^**(CH_3_COO)_2_]^2+^.

#### 2.2.2. Synthesis of Cu_2_**L^4^**

In an inert atmosphere glove box, **L^4^** (0.555 g, 1 mmol) and anhydrous copper(II) acetate (0.363 g, 2 mmol) were dissolved in methanol (30 mL) and stirred for 1 day. The resulting blue solution was removed from the glovebox and filtered to remove trace solids. NH_4_PF_6_ (5 equiv.) was dissolved in methanol (10 mL) and added to the copper complex. Precipitation occurred immediately and was completed by standing in a freezer overnight. The blue powder was filtered and washed with methanol and diethyl ether, yielding Cu_2_**L^4^** (0.360 g, 30%). Elemental analysis: calculated (%) for [C_32_H_58_N_8_Cu_2_](CH_3_COO)_1.5_(PF_6_)_2.5_•1.5CH_3_OH: C, 37.12; H, 5.85; N, 9.49. Found: C, 37.38; H, 5.85; N, 9.18. MS (ESI): *m*/*z* = 770 [HCu**L^4^**(CH_3_COO)_2_(CH_3_OH)]^+^, *m*/*z* = 400 [Cu_2_**L^4^**(CH_3_COO)_2_]^2+^.

#### 2.2.3. Synthesis of Zn_2_**L^4^**

In an inert atmosphere glove box, **L^4^** (0.555 g, 1 mmol) and anhydrous zinc(II) acetate (0.367 g, 2 mmol) were dissolved in methanol (30 mL) and stirred for 1 day. The resulting yellow solution was removed from the glovebox and filtered to remove trace solids. NH_4_PF_6_ (5 equiv.) was dissolved in methanol (10 mL) and added to the zinc complex. Precipitation occurred immediately and was completed by standing in a freezer overnight. The powder was filtered, yielding Zn_2_**L^4^** (0.443 g, 41%). Elemental analysis: calculated (%) for [C_30_H_58_N_8_Zn_2_](CH_3_COO)_2.3_(PF_6_)_1.7_•1H_2_O: C, 40.48; H, 6.21; N, 10.32. Found: C, 40.15; H, 6.54; N, 10.62. MS (ESI): *m*/*z* = 402 [Zn_2_**L^4^**(CH_3_COO)_2_]^2+^.

#### 2.2.4. Synthesis of Cu_2_**L^5^**

In an inert atmosphere glove box, **L^5^** (0.527 g, 1 mmol) and anhydrous copper(II) acetate (0.363 g, 2 mmol) were dissolved in acetonitrile (30 mL) and stirred for 1 day. The solvent was evaporated, yielding a blue oil. The blue residue was dissolved in methanol (10 mL). NH_4_PF_6_ (5 equiv.) was dissolved in methanol (10 mL) and added to the copper complex. Precipitation occurred immediately and was completed by standing in a freezer overnight. The blue powder was filtered and washed with methanol and diethyl ether, yielding Cu_2_**L^5^** (1.06 g, 89%). Elemental analysis: calculated (%) for [C_30_H_54_N_8_Cu_2_](CH_3_COO)_2_(PF_6_)_2_•0.8NH_4_PF_6_: C, 34.25; H, 5.34; N, 10.34. Found: C, 34.25; H, 5.18; N 9.95. MS (ESI): *m*/*z* = 386 [Cu_2_**L^5^**(CH_3_COO)_2_]^2+^.

#### 2.2.5. Synthesis of Zn_2_**L^5^**

In an inert atmosphere glove box, **L^5^** (0.527 g, 1 mmol) and anhydrous zinc(II) acetate (0.367 g, 2 mmol) were dissolved in acetonitrile (30 mL) and stirred for 1 day. The solvent was evaporated, yielding a yellow oil. The residue was dissolved in methanol (10 mL). NH_4_PF_6_ (5 equiv.) was dissolved in methanol (10 mL) and added to the zinc complex. Precipitation occurred immediately and was completed by standing in a freezer overnight. The powder was filtered and washed with methanol and diethyl ether, yielding Zn_2_**L^5^** (1.06 g, 98%). Elemental analysis: calculated (%) for [C_30_H_54_N_8_Zn_2_](CH_3_COO)_2_(PF_6_)_2_•1H_2_O: C, 37.68; H, 5.77; N, 10.34. Found: C, 37.79; H, 5.77; N, 10.20. MS (ESI): *m*/*z* = 388 [Zn_2_**L^5^**(CH_3_COO)_2_]^2+^.

### 2.3. Biological Evaluation

#### 2.3.1. General

Buffy coat preparations from healthy donors were obtained from the Blood Bank in Leuven, Belgium. For the chemokine binding studies, peripheral blood mononuclear cells (PBMCs) activated with phytohemagglutinin (PHA; Sigma, Belgium) were used. Human T-lymphocytic SUP-T1 cells and TZM-bl cells were obtained from the American Type Culture Collection (Rockville, MD) and maintained in RPMI-1640 supplemented with 10% foetal bovine serum (FBS). Human glioblastoma U87.MG and U87.CD4 cells were kindly provided by Dr. D. Littman [46,47] and used to generate cells expressing CXCR4 or CCR5 [48]. The fluorescent chemokines CXCL12^AF647^ and CCL5^AF647^ were purchased from Almac. The CXCR4 inhibitor AMD3100 and the CCR5 inhibitor maraviroc were a kind gift of Dr. Gary Bridger, affiliated at that time with AnorMED (Langley, Canada). HIV-1 NL4-3 (CXCR4-using, X4) and HIV-1 BaL (CCR5-using, R5) were obtained through the AIDS Research and Reference Reagent Program (Division of AIDS, NIAID, NIH).

#### 2.3.2. HIV Infection Assay

TZM-bl cells [49] were seeded in transparent 96-well plates at 1 × 10^4^ cells per well in DMEM (Dulbecco’s modified Eagle medium; Life Technologies, Waltham, MA, USA) with 10% FBS and 10 mM HEPES. Subsequently, compounds were added, and the cell/sample mixture was incubated at 37 °C. After 30 min, the virus was added at 100 pg p-24 per well. After 48 h of incubation, the assay plates were analysed. For the analysis, steadylite plus substrate solution (PerkinElmer, Waltham, MA, USA) was added to the assay plates. The luminescent signal of the lysed cell suspension was analysed in white 96-well plates on a Spectra-Max L luminescence microplate reader (Molecular Devices, Sunnyvale, CA, USA) after a 10 min incubation period in the dark. Luciferase activity induced by HIV-1 Tat protein expression was measured as an assessment of the amount of HIV replication.

#### 2.3.3. Anti-CXCR4 Antibody (Clone 12G5) Binding Inhibition Assay

SUP-T1 cells were washed in phosphate buffered saline (PBS) with 2% FBS and resuspended at 3 × 10^6^ cells/mL in the same buffer solution. For each sample, 0.3 × 10^6^ cells were pre-incubated with the sample in PBS/FBS 2% for 30 min at 4 °C. The cells were washed once in PBS/FBS 2% before the addition of the monoclonal antibody (mAb) against CXCR4 (clone 12G5; BD Biosciences, San Jose, CA, USA). After incubation (30 min at 4 °C), the cells were washed, resuspended in PBS containing 1% paraformaldehyde, and analysed on a FACSCalibur flow cytometer (Becton Dickinson, San Jose, CA, USA).

#### 2.3.4. Chemokine (CXCL12^AF647^/CCL5^AF647^) Binding Inhibition Assay

Human PBMCs were washed once with assay buffer (Hanks’ balanced salt solution with 20 mM HEPES buffer and 0.2% bovine serum albumin, pH 7.4) and then incubated for 15 min at room temperature with the sample diluted in assay buffer at the indicated concentrations. Subsequently, CXCL12^AF647^ (25 ng/mL) or CCL5^AF647^ (50 ng/mL) was added to the compound-incubated cells. The cells were incubated for 30 min at room temperature. Thereafter, the cells were washed twice in assay buffer, fixed in 1% paraformaldehyde in PBS, and analysed on the FL4 channel of a FACSCalibur flow cytometer equipped with a 635 nm red diode laser (Becton Dickinson, San Jose, CA, USA). The percentage of inhibition of CXCL12^AF647^ or CCL5^AF647^ binding was calculated according to the formula: [1 − ((MFI − MFI_NC_)/(MFI_PC_ − MFI_NC_))] × 100, where MFI is the mean fluorescence intensity of the cells incubated with CXCL12^AF647^ or CCL5^AF647^ in the presence of the inhibitor, MFI_NC_ is the mean fluorescence intensity measured in the negative control (i.e., autofluorescence of unlabelled cells), and MFI_PC_ is the mean fluorescence intensity of the positive control (i.e., cells exposed to CXCL12^AF647^ or CCL5^AF647^ alone).

#### 2.3.5. Measurement of Intracellular Chemokine-Induced Calcium Mobilisation

CXCR4- or CCR5-positive U87 glioblastoma/astrocytoma cells were loaded with the fluorescent calcium indicator Fluo-3 acetoxymethyl (Molecular Probes) at 4 µM in the assay buffer (Hanks’ balanced salt solution with 20 mM HEPES buffer and 0.2% bovine serum albumin, pH 7.4) for 45 min at room temperature. After thorough washing with the assay buffer, cells were pre-incubated for 10 min at 37 °C in the same buffer with the compounds of interest. The intracellular calcium mobilisation in response to CXCL12 (CXCR4 chemokine ligand) or CCL3L1 (CCR5 chemokine ligand) was then measured at 37 °C by monitoring the fluorescence as a function of time simultaneously in all the wells by using a fluorometric imaging plate reader (FLIPR; Molecular Devices, Sunnyvale, CA, USA).

#### 2.3.6. Evaluation of Cellular Cytotoxicity

Cytotoxicity of the compounds was evaluated after 48 h of incubation at 37 °C in PHA-stimulated PBMCs (5 × 10^5^ cells per well in culture medium). Each condition was tested in duplicate. Cytotoxicity was evaluated microscopically, and in addition, the cell viability was assessed using the colorimetric CellTiter 96 AQueous One Solution Cell Proliferation Assay (Promega, Fitchburg, WI, USA), which is based on the reduction of the tetrazolium salt MTS [3-(4,5-dimethylthiazol-2-yl)-5-(3-carboxymethoxyphenyl)-2-(4-sulfophenyl)-2H-tetrazolium] to formazan by metabolically active cells. Absorbance at 490 nm was measured with the Versa-Max ELISA microplate reader (Molecular Devices, Sunnyvale, CA, USA). The cytotoxic concentration 50 (CC_50_) of each compound was calculated based on the absorbance of negative (i.e., cells without compound) and positive (i.e., only culture medium) control samples.

### 2.4. X-ray Crystallography

Single-crystal X-ray diffraction data were collected in a series of ω-scans using a Stoe IPSD2 image plate diffractometer utilising monochromated Mo radiation (λ = 0.71073 Å). Standard procedures were employed for the integration and processing of the data using X-RED [50]. Samples were coated in a thin film of perfluoro-polyether oil and mounted at the tip of a glass fibre located on a goniometer. Data were collected from crystals held at 150 K in an Oxford Cryosystems nitrogen gas cryostream. Crystal structures were solved using routine automatic direct methods implemented within SHELXS-97 [51]. Completion of structures was achieved by performing least-squares refinement against all unique F^2^ values using SHELXL2018 [51]. All non-H atoms were refined with anisotropic displacement parameters. Hydrogen atoms were placed using a riding model. Disorder, where present, was treated conservatively with standard methods. In each structure, the core was well-determined, but the unbound anions were much less well-determined. Further information is provided in the Supplementary Materials.

### 2.5. Computational Methods

Single macrocycle components of the Zn(II) and Cu(II) complexes of **sL^1^**–**sL^5^**, with water and/or acetate bound, were computationally modelled to obtain an estimate of the thermodynamics of binding. All quantum mechanical calculations were performed using *Gaussian 09* software (*RevA.02*) [52]. Density functional theory (DFT) calculations were performed utilising the M06 functional with an ultrafine integration grid, and the all electron cc-pVDZ basis set was used on all atoms [53]. Full geometry optimisations and vibrational frequency calculations were performed in the gas phase. All calculations of the Zn(II) complexes were run in the singlet electronic state, while the Cu(II) complexes were run as doublet states.

## 3. Results and Discussion

In previous studies, we characterised copper(II) and zinc(II) complexes of **L^2^** (Figure 1) and showed that the zinc(II) complex is a more potent CXCR4 antagonist than the copper(II) complex. A single configuration of the cyclam ring (*trans*-II) was observed in both the solid state and solution (using NMR) for [Zn_2_**L^2^**]^4+^, and so the potency could be correlated with the coordination sphere around the metal centre [33]. X-ray crystallography, DFT computational studies, and in vitro analyses of the biological properties have shown that the weaker bonding for [Cu_2_**L^2^**]^4+^ at the “free” coordination site for protein binding reduces potency [43]. We then reported the bis-cross-bridged cyclam derivative **L^3^** (Figure 1) and its copper(II) complex, which showed an increase in rigidity and higher affinity for binding to the aspartate residues at the extracellular surface of the CXCR4 chemokine receptor than [Cu_2_**L^2^**]^4+^ (Table 1 and Table 2) [34]. The complexes [Zn_2_**L^2^**]^4+^ and [Cu_2_**L^3^**]^4+^ were identified as the lead compounds from our previous studies, showing higher affinity and increased residence time in vitro relative to non-bridged compounds. To complete the study and provide a more complete understanding of the key molecular properties, the [Zn_2_**L^3^**]^4+^ complex was synthesised along with cross-bridged derivatives where the ring size was varied (**L^4^** and **L^5^**) to determine the impact of cavity size on the binding affinity, biological activity, and receptor selectivity. Further X-ray structural characterisation of [Cu_2_**L^2^**]^4+^ is also presented. The aim is to optimise the coordination interactions and the affinity for the CXCR4 receptor to improve the pharmacological profile of this class of compounds.

### 3.1. Synthesis of Ligands and Metal Complexes

**Scheme 1:**
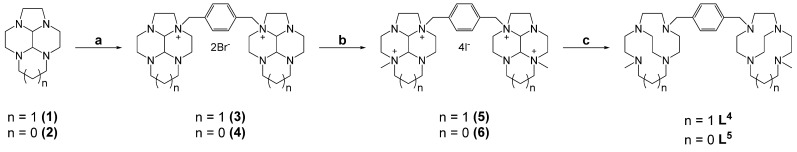
Synthesis of para-linked bis-tetraazamacrocycles homocyclen (**L^4^**) and cyclen (**L^5^**). (**a**) *p*-Dibromoxylene; (**b**) CH_3_I; (**c**) NaBH_4_.

The synthesis of the novel chelators **L^4^** and **L^5^** and their copper(II) and zinc(II) complexes followed our previously reported route for the synthesis of the chelator **L^3^** and were achieved on a multi-gram scale with all steps with a >70% yield. We have previously reported the synthesis of *para*-substituted ethyl-bridged cyclams [34]. The synthetic methods were applied to the 13-membered tetraazamacrocycle homocyclen to form **L^4^** and the 12-membered cyclen to form **L^5^**. The ligands were formed by reacting the macrocycle glyoxal condensates (**1** and **2**) with α,α’-dibromo-*p*-xylene to form bis-tetraazamacrocycles **3** and **4**. The intermediates were subsequently alkylated with methyl iodide (**5** and **6**), followed by reduction with sodium borohydride to form the target chelators (Scheme 1). Copper(II) and zinc(II) complexes were formed in an analogous method to that described previously, in methanol and using acetate salts of the metal ions [34].

### 3.2. Crystallographic and Spectroscopic Characterisation of Copper(II) Complexes

**Figure 2:**
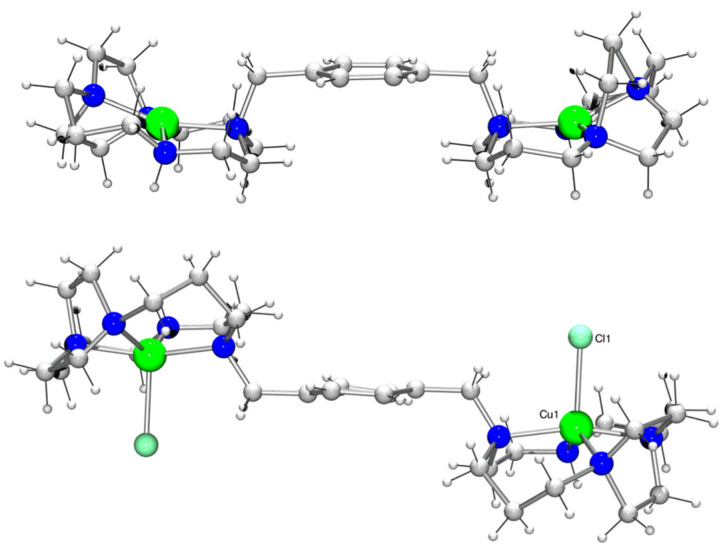
X-ray crystal structures of [Cu_2_**L^2^**](ClO_4_)_4_ (**top**) and [Cu_2_**L^2^**Cl_2_]Cl_2_ (**bottom**), non-coordinating anions omitted for clarity (see Supplementary Materials for further details and the ORTEP plots).

Single crystals of both [Cu_2_**L^2^**](ClO_4_)_4_ and [Cu_2_**L^2^**Cl_2_]Cl_2_ (Figure 2) were grown via ether diffusion into a methanolic solution. The crystal structure of [Cu_2_**L^2^**](ClO_4_)_4_ contained two macrocyclic units and was in a *trans*-II configuration. The copper(II) ions in each ring were not crystallographically equivalent to each other, but their environments were very similar. They both adopted a tetrahedrally distorted square planar geometry, with Cu−N bond lengths ranging from 1.879(14) to 2.073(17) Å in one ring and 1.871(16) to 2.237(15) Å in the second. The metal–nitrogen bond lengths in [Cu_2_**L^2^**]^4+^ were, as expected, somewhat shorter than in [Zn_2_**L^2^**(OAc)_2_]^2+^, and in addition, there was no significant interaction between the copper(II) centre and the counter anion. The Cu(1)…O(16) and Cu(2)…O(5) distances were 3.16(2) and 2.58(2) Å, respectively (Figure S1 and Tables S1–S3). An example of a mono-side-bridged copper(II) complex reported by Kaden and co-workers had two independent copper(II) environments in the asymmetric unit [54]. The Cu−N bond lengths were similar to [Cu_2_**L^2^**]^4+^, being in the range of 1.979(4) to 2.016(4) Å. Analogous to [Cu_2_**L^2^**]^4+^, the copper(II) was also four-coordinate in the solid state. The macrocyclic framework was in a *trans*-II configuration, where the methyl and hydrogen arms sat *trans* to each other. In each of the two crystal structures, the copper(II) ion sat in the plane of the four nitrogen atoms, the deviation from the mean plane being 0.070 and 0.007 Å, respectively.

The analysis of the crystal structure of [Cu_2_**L^2^**Cl_2_]^2+^ was complicated because the asymmetric unit lay on a crystallographic mirror plane that did not correspond with a mirror plane in the molecule (Figure 2). This generated two orientations for the molecule in equal amounts. The copper(II) centre was five-coordinate and can best be described as being in a square-based pyramidal coordination environment, the base consisting of the four macrocyclic nitrogen atoms with a bound chloride counter-anion in the apical position (Cu(1)…Cl(1) distance is 2.4133(14) Å). The copper(II)–nitrogen bond lengths were in the range of 1.938(7) to 2.141(6) Å (Figure S2 and Tables S4–S6), which was similar to [Cu_2_**L^2^**]^4+^. Again, the macrocycle adopted the equivalent of a *trans*-II configuration in the solid state (Figure 2). The metal–metal distance of 11.5637(14) Å was, as expected, longer than in [Cu_2_**L^2^**]^4+^ (10.913(2) Å in [Cu_2_**L^2^**]^4+^), as the two macrocycles were located in the equivalent of a *trans* position relative to each other. In the structure of [Cu_2_**L^2^**](ClO_4_)_4_, the metal centres were *cis* to each other with a three point angle of 151.31(9)°, whereas in [Cu_2_**L^2^**Cl_2_]Cl_2_, the metal centres were *trans* to each other with a three point angle of exactly 180° due to the crystallographic inversion centre. This distance was similar to that found in [Zn_2_**L^2^**(OAc)_2_]^2+^ (11.6584(10) Å) [33]. The angle between the two macrocycles could then be determined. The same three planes were defined and resulted in an angle of *ca.* 180°, similar to [Zn_2_**L^2^**(OAc)_2_]^2+^, with any deviation being attributed to the disorder in the structure. The angle between the plane of the carbon–nitrogen bond and the plane of the phenyl moiety was 88.200(99)°.

UV/Vis was used to investigate the solution behaviour of [Cu_2_**L^2^**]^4+^, showing a broad band at 557 nm [43]. This is a higher value than that usually observed for square planar copper(II) cyclam complexes, which can be attributed to the tetragonal distortion of the copper(II) centre. A more appropriate comparison is with the mono-side-bridged cyclam copper complex reported by Kowallick et al., which had an absorption maximum at 556 nm in aqueous solution [54]. Five-coordinate copper(II) complexes generally exhibit a band at around 600 nm [55]. There was no significant shift in the band at 557 nm upon the addition of sodium acetate (2 M sodium acetate; 560 nm), suggesting a preference for a four-coordinate copper(II) species in solution.

### 3.3. DFT Calculations

**Figure 3:**
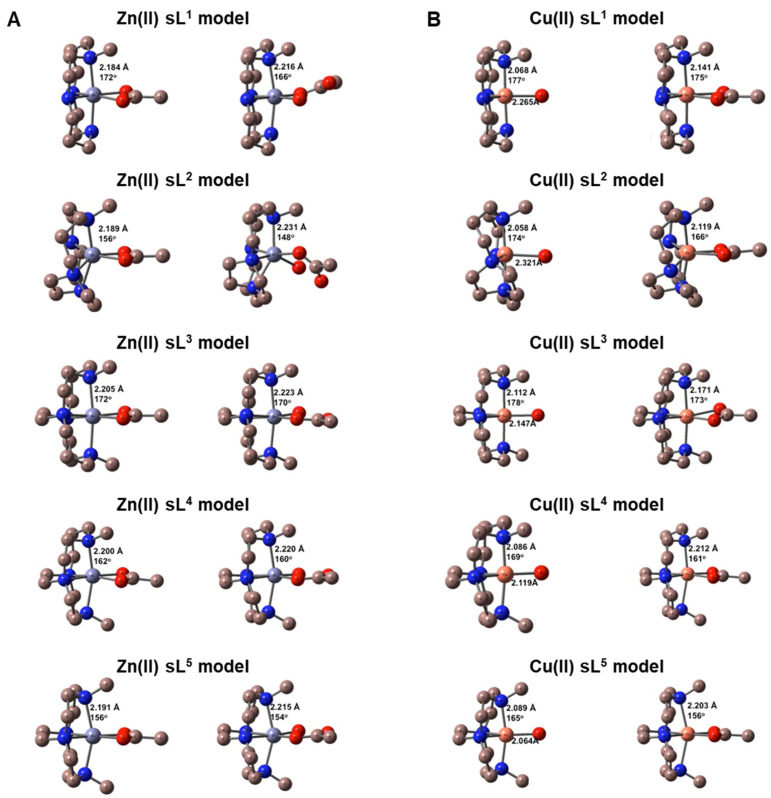
DFT calculated structures of single ring models of the macrocyclic complexes with zinc(II) and copper(II). (**A**) [Zn**sL**(OAc)]^+^ on the left and [Zn**sL**(OAc)(H_2_O)]^+^ on the right. Values shown are the average Zn–N bond lengths and the axial N–Zn–N angle. (**B**) [Cu**sL**(H_2_O)]^2+^ on the left and [Cu**sL**(OAc)]^+^ on the right. Values shown are the average Cu–N bond lengths, the axial N–Cu–N angle, and the Cu–O bond length.

To obtain an estimate of the strength of water and/or acetate binding to the zinc(II) and copper(II) complexes of **L^1^** through **L^5^**, DFT calculations were performed on single ring models of these metal complexes, which had the chelators designated as **sL^1^** through **sL^5^** (Figure 3). It was anticipated that increased affinity for acetate coordination over a water molecule would correspond to increased receptor affinity. This mimics the coordination interaction with aspartate or glutamate on the chemokine receptor protein surface.

M06/cc-pVDZ calculations were performed on all complexes. Figure 3A shows the DFT geometry optimised structures for [Zn**sL**(OAc)]^+^ on the left and [Zn**sL**(OAc)(H_2_O)]^+^ on the right ([Zn**sL**(H_2_O)_2_]^2+^ data are shown in Figure S3), while Figure 3B shows the structures for [Cu**sL**(H_2_O)]^2+^ on the left and [Cu**sL**(OAc)]^+^ on the right. In each of these figures, the bonding parameters listed are the average M(II)–N bond lengths in angstroms (Å) and the axial N–M(II)–N angle in degrees (°). Based on our previous studies, zinc(II) complexes tend to have uniformly high activity in biological assays, and assuming that the two metal ions independently form coordinate bonds with different amino acid residues on the extracellular surface of CXCR4, single ring complexes were modelled. For the zinc(II) complex, there was particular interest in the influence of the macrocycle ring size on the inclusion of a water molecule along with the monodentate acetate in the coordination sphere. In correlation with the X-ray structural data that have previously been reported, a strong preference for the inclusion of a water molecule in the coordination sphere was observed for the cross-bridged complexes [56]. For zinc(II), it was more favourable to have the water molecule present in all of the structures, except for the side-bridged macrocyclic chelator present in **sL^2^**, which was more stable with the bidentate acetate present. The Gibbs free energies of binding, as listed in Table 3, specify the order of interaction strength as: **sL^3^** > **sL^4^** > **sL^5^** > **sL^1^** > **sL^2^**. This is consistent with the X-ray dataset previously reported [56].

Similarly for the copper(II) complexes, the optimised structures were analogous to the reported crystal structures of related complexes. The [Cu**sL**(H_2_O)]^2+^ complexes were five-coordinate while the [Cu**sL**(OAc)]^+^ complexes were six-coordinate, with the two oxygen atoms of the coordinated acetate occupying *cis* equatorial positions in an anisotropic bidentate fashion for **sL^1^**, **sL^2^**, and **sL^3^**, while the two Cu(II)–O bond lengths were similar in the **sL^4^** and **sL^5^** complexes. It was favourable for the acetate anion to replace the coordinated water molecule in all complexes with the Gibbs energy changes listed in Table 3. However, similar to the zinc(II) complexes, the **sL^2^** ligand had the least favourable reaction energy, with the order of interaction being: **sL^5^** > **sL^4^** > **sL^3^** > **sL^1^** > **sL^2^**. Interestingly, the acetate binding energies tracked the Cu(II)–OH_2_ bond length: as Cu(II)–O increased in [Cu**sL**(H_2_O)]^2+^, the acetate binding strength decreased. The Cu(II)–O values are also shown in Figure 3B. The calculations for copper(II) were designed to determine whether the acetate interaction correlated to the affinity recorded in the biological assays. Binding of the acetate over water was strongly preferred in all cases. The copper(II) complex of the side-bridged macrocycle (**sL^2^**) in [Cu_2_**L^2^**]^4+^ had the lowest binding energy for acetate, which matched with both the reduced potency of this CXCR4 antagonist and the UV/Vis solution studies. The calculations validated the observed increase in affinity for the cross-bridged compounds (Table 1 and Table 2). There was a slight increase in the binding energy for acetate as the ring size decreased, with the most favourable reaction for acetate in replacing a bound water observed for cross-bridged cyclen **sL^5^** (Table 3). In general, these calculations indicate that [Cu_2_**L^5^**]^4+^ should have the most favourable interaction with the CXCR4 receptor, although there were no major differences between the three cross-bridged derivatives for this parameter.

### 3.4. Biological Evaluation

A series of binding assays were performed to assess the in vitro biological activity and function of the small molecules targeting CXCR4. Data demonstrating HIV-1-CXCR4 entry blocking, interference with the CXCL12-CXCR4 axis, anti-CXCR4 antibody competition, and receptor specificity are shown in Table 1 and Table 2.

#### 3.4.1. Assessment of Cellular Cytotoxicity for the Investigated Compounds

The cellular cytotoxicity of all compounds was evaluated in PHA-stimulated PBMCs. The cytotoxic concentration 50 (i.e., CC_50_; the concentration that reduces cell viability by 50%) was determined, based on the microscopic evaluation of the PBMCs and quantification of their cell viability using the MTS assay. CC_50_ values were close to or higher than 100 µM for most of the investigated metal complexes (Cu_2_**L^1^**, Zn_2_**L^1^**, Cu_2_**L^2^**, Zn_2_**L^2^**, Zn_2_**L^3^**, Zn_2_**L^4^**, and Zn_2_**L^5^**) as well as for the reference compounds AMD3100 and Maraviroc. Cu_2_**L^4^** had the lowest CC_50_ value in PBMCs (>10 µM). Furthermore, CC_50_ values of the copper(II) complexes of **L^3^** and **L^5^** were moderate to high, situated around 50 to 40 µM, respectively (Table 1). The CC_50_ values indicate low toxicity in relation to the anticipated dosing levels.

#### 3.4.2. Antiviral Activity against HIV-1 NL4-3 (X4) and BaL (R5) Replication

CXCR4 is one of the two main co-receptors involved in the infection process of HIV-1 [57,58]. In an initial set of experiments, the compounds were evaluated for their anti-HIV-1 activity. The effect of the compounds on the replication of the CXCR4-using HIV-1 strain NL4-3 (X4) and the CCR5-using HIV-1 strain BaL (R5) was determined in a HIV Tat-regulated luciferase reporter gene assay in the TZM-bl cells. IC_50_ values for the inhibition of the HIV-1 strain NL4-3 (X4) were in the low nanomolar range (0.73–31.6 nM). Inhibition of the HIV-1 strain BaL (R5) was less efficient, with IC_50_ values varying greater than 1 µM (Table 1), highlighting the specificity of the different derivatives to the CXCR4 receptor. IC_50_ values of AMD3100, a CXCR4-specific inhibitor, and Maraviroc, a CCR5-specific inhibitor, were 0.42 ± 0.14 nM and 2.9 ± 1.2 nM for X4 and R5 HIV-1 replication inhibition, respectively (Table 1). Efficient inhibition of CXCR4-using (X4) HIV-1 was observed for all compounds, with a lower potency observed for the copper(II) complexes of **L^1^**, **L^2^**, and **L^5^** compared to their zinc(II) counterparts. In contrast, a higher potency for the copper(II) cross-bridged complexes (**L^3^** and **L^4^**) was observed compared to the corresponding zinc(II) complexes. The least active compound (Cu_2_**L^5^**) had an IC_50_ value of 31.6 ± 2.4 nM. None of the complexes exhibited any increase in anti-HIV activity compared to AMD3100. All metal complexes showed weak, if any, activity against the R5 HIV-1 strain (IC_50_ > 1 µM), suggesting selectivity for CXCR4 over CCR5 (Table 1).

#### 3.4.3. Inhibition of Anti-CXCR4 Monoclonal Antibody (Clone 12G5) Binding

The inhibitory potency of the compounds was further evaluated through competition binding experiments using the anti-CXCR4 monoclonal antibody (clone 12G5). The 12G5 antibody has been reported to block the entry process of HIV-1 via CXCR4 [59]. Successful blocking of the binding of the 12G5 antibody to CXCR4 using the small molecule inhibitor AMD3100 has previously been reported [60], confirming that this antibody can be a useful tool to study the interactions of the novel metal complexes reported herein with CXCR4. The results are shown in Table 2 and Figure S4. All of the compounds have promising efficacy as CXCR4 inhibitors, with IC_50_ values in the nanomolar range, ranging from 465 nM to 1.12 nM. Amongst the different metal complexes, Zn_2_**L^2^**, Cu_2_**L^3^**, Zn_2_**L^3^**, Cu_2_**L^4^**, and Cu_2_**L^5^** showed the highest potency with IC_50_ values < 2 nM. (Table 2).

#### 3.4.4. Potent Antagonism of Natural Chemokine Ligand Interaction to CXCR4 and Receptor Selectivity Against CCR5

Binding affinity and selectivity towards CXCR4 were further evaluated through competition binding assays using natural chemokine ligands, tagged with Alexa Fluor 647, as previously reported [61,62]. The CXCR4 binding affinity of the different compounds was determined through their ability to inhibit interactions between CXCR4 and CXCL12^AF647^. Selectivity towards CXCR4 was evaluated in a similar assay using CCR5, another co-receptor involved in HIV entry mechanism, and its fluorescent-labelled natural ligand CCL5^AF647^. All of the metal complexes showed a dose-dependent inhibition of CXCL12^AF647^ binding to CXCR4, with low nanomolar IC_50_ values ranging from 0.41 to 8.9 nM (Table 2 and Figure S5). Interestingly, apart from Zn_2_**L^4^** (2.1 ± 0.1 nM), all of the other zinc(II) complexes had very similar high potency (<1 nM). The reference CXCR4 inhibitor, AMD3100, was less effective, with an IC_50_ value of 18.0 ± 4.2 nM (Table 2). In competition binding experiments using CCR5/CCL5^AF647^, IC_50_ values > 1 µM were observed for all but three compounds (Zn_2_**L^2^**, Cu_2_**L^3^**, Cu_2_**L^4^**), indicating selectivity towards CXCR4. Although lower, the IC_50_ of Zn_2_**L^2^**, Cu_2_**L^3^** and Cu_2_**L^4^** still remained in the medium-high nanomolar range with values of 255 ± 58 nM, 741 ± 145 nM, and 702 ± 219 nM, respectively (Table 2 and Figure S5). As a reference, the CCR5-specific inhibitor, Maraviroc, displayed an IC_50_ value of 10.3 ± 3.5 nM in this assay (Table 2). In this competition binding assay using CXCL12^AF647^, variation in the ring size had more of an effect on the copper(II) complexes in comparison with the zinc(II) complexes, which, apart from Zn_2_**L^4^**, displayed similar activity (Zn_2_**L^1^**, Zn_2_**L^2^**, Zn_2_**L^3^**, Zn_2_**L^5^**). Interestingly, Zn_2_**L^3^**, Zn_2_**L^4^**, and Zn_2_**L^5^** displayed improved selectivity for CXCR4 (>1 µM for CCR5) compared to the previous lead compound Zn_2_**L^2^** (255 ± 58 nM for CCR5). All metal complexes studied displayed higher activity compared to AMD3100.

#### 3.4.5. Inhibition of Chemokine Induced Signalling via CXCR4 or CCR5

**Figure 4:**
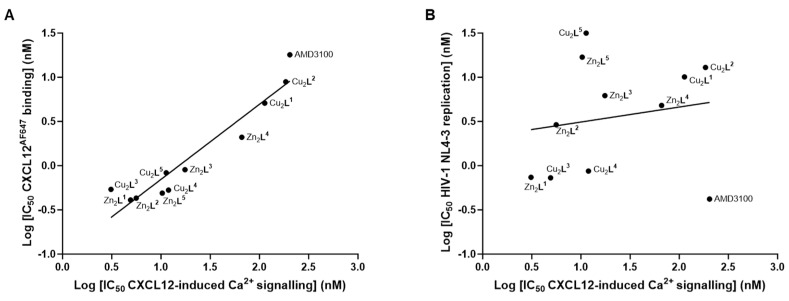
Correlation of the inhibitory effects of the experimental compounds regarding CXCL12-induced calcium signalling with CXCL12^AF647^ binding (correlation coefficient r = 0.95) (**A**) or HIV-1 NL4-3 (X4) replication (correlation coefficient r = 0.17) (**B**).

The potency of the different metal complexes was investigated in a functional downstream receptor signalling assay. The inhibitory effect of the tested compounds was examined by Ca^2+^ mobilisation studies with the natural ligands CXCL12 and CCL3L1 for the chemokine receptors CXCR4 and CCR5, respectively. The Ca^2+^ ions released from the endoplasmic reticulum were measured over time in CXCR4- or CCR5-transfected U87 glioblastoma cells via their specific interaction with the fluorescent Ca^2+^ indicator Fluo-3 [63]. The IC_50_ values measured for the inhibition of CXCL12-CXCR4 signalling were in the low nanomolar range (3.1–185 nM), and all compounds were more potent than AMD3100 (203.5 ± 19.4 nM) in this assay (Table 2). In the case of CCR5, IC_50_ values measured were all in the micromolar range, except for Zn_2_**L^2^** (120 ± 28 nM), which appeared to be acting as a dual antagonist of CXCR4 and CCR5 in this experimental setup (Table 2). A strong correlation was seen between the IC_50_ values of CXCL12-induced calcium signalling via CXCR4 and CXCL12-CXCR4 binding (Figure 4A).

In this receptor functional assay (CXCL12-induced calcium signalling via CXCR4), varying the ring size of the copper(II) complexes (**L^4^**/**L^5^**) did not impact significantly on the high affinity for CXCR4 compared to the previous lead compound Cu_2_**L^3^**; however, it resulted in a 10-fold increase in selectivity for CXCR4 over CCR5. Similar results were observed with Zn_2_**L^5^**, which retained a high affinity for CXCR4 (10.3 ± 6.6 nM) compared to the previous Zn_2_**L^2^** (5.6 ± 1.9 nM) lead candidate, but with a >100-fold increase in selectivity for CXCR4 (Table 2). Zn_2_**L^2^** was the only compound with a sub-micromolar activity in the CCL3L1-induced calcium signalling assay, with an IC_50_ value of 120 ± 28 nM (Table 2). The latter observation could imply that significant off-target effects will be observed if using the Zn_2_**L^2^** compound as a prognostic tracer or therapy for CXCR4 overexpressing cells in vivo. A correlation plot showed consistency between the CXCR4 chemokine binding assay and calcium signalling assay (r = 0.95, Figure 4A). However, no correlation was found between the CXCL12-induced calcium signalling assay and anti-HIV properties (r = 0.17, Figure 4B), indicating that an alternate optimisation route is required to develop antiviral compounds over chemokine signal blocking compounds. Further correlation plots are available in Figure S6.

## 4. Conclusions

Structural investigation of the influence of macrocycle ring size on a range of metal ion complexes demonstrated that in similar compounds, subtle effects on the coordination geometry and positioning of the metal ion in the cavity dictate the biological efficacy and target selectivity.

AMD3100 effectively inhibited HIV infection in vitro, but was inferior for blocking the CXCL12 signalling pathway, which is relevant to the growth and dissemination of multiple cancers. All of the metal complexes investigated in this work compared favourably with AMD3100, with Cu_2_**L^2^** having an overall similar profile. The advantage of configurational restriction is clear from a coordination geometry perspective, especially with the copper(II) complexes, as the cross-bridged compounds had much higher activity than the unrestricted or side-bridged compounds. The flexible coordination geometry of the d^10^ zinc(II) ion showed little difference in the compound potency across the zinc(II) compounds tested, with all showing significant improvement over AMD3100. However, the zinc(II) complexes of **L^3^**, **L^4^**, and **L^5^** also showed a significant improvement in the selectivity for CXCR4 (over CCR5) whilst still retaining a similar high affinity and activity against CXCL12 binding compared to previous lead compounds. This is important for drug development progression where selectivity is as important as affinity/activity. Based on the observations reported in the study, with high importance placed on the binding selectivity, we identified Zn_2_**L^5^** as a new drug candidate to take forward to target the CXCL12–CXCR4 axis in cancer therapy studies. We also plan to continue with our investigation and development of Cu_2_**L^3^** in cancer imaging and therapy. It is noteworthy that zinc(II) metallodrugs have applications as cancer therapeutics alongside other medicinal applications [64].

Whilst the trend observed in the calculations can be rationalised (as the metal ion is pushed out of the ring, it will interact more strongly with the acetate/aspartate residues), it is not consistent with the biological assay results. This is likely due to factors such as the relative positioning of the two metal centres for optimum interactions, and possibly, the reduced stability of the copper(II) complexes of cross-bridged homocyclen and cyclen. Zinc(II) and copper(II) are ideal comparators as the d^9^ copper(II) ion has distinct geometric preferences (and stabilises distortion for octahedral and square-based pyramidal geometry), whereas the d^10^ zinc(II) ion is more flexible in the coordination sphere that can be adopted. This was reflected in the assay results. However, the optimised copper(II) complexes could be radiolabelled with copper-64 (PET imaging radioisotope) or copper-67 (therapeutic/SPECT imaging radioisotope), offering a route to the development of CXCR4-targeted theranostics for cancer imaging and therapy. In addition to the biological assay data, it has previously been shown that complex stability for copper(II) is higher for the cross-bridged cyclam compared to the cross-bridged homocyclen and cross-bridged cyclen [36], suggesting that the metal ion would be more likely to be retained in vivo. This is in line with our previously reported data showing that the configuration of Cu_2_**L^3^** leads to increased affinity for CXCR4, stronger interactions, and longer residence time at the receptor [34]. We have also recently shown that **L^3^** can be successfully and stably radiolabelled with copper-64, resulting in high and specific CXCR4-mediated tumour uptake [23]. Due to the improved selectivity observed, further consideration should be given for the radiolabelling and stability evaluation of Cu_2_**L^5^** to determine whether this complex could become a candidate for theranostic development. For applications targeting the CXCL12–CXCR4 signalling axis in cancer therapy, Zn_2_**L^5^** provides a further option for comparison to our current lead compound, Cu_2_**L^3^**.

## Figures and Tables

**Table 1:** Anti-HIV and cellular toxicity evaluation of CXCR4 antagonists.

Compound	Anti-HIV Activity ^a^ IC_50_ (nM)		Toxicity ^b^ CC_50_ (µM)
	NL4-3 (X4)	BaL (R5)	PMBCs
Cu_2_**L^1^**	10.1 ± 1.6	>20,000	>100
Zn_2_**L^1^**	0.73 ± 0.34	>15,000	>90
Cu_2_**L^2^**	12.9 ± 2.8	>20,000	>100
Zn_2_**L^2^**	2.9 ± 1.2	>15,000	>100
Cu_2_**L^3^**	0.74 ± 0.60	>5000	>40
Zn_2_**L^3^**	6.2 ± 2.5	>1000	>100
Cu_2_**L^4^**	0.87 ± 0.32	>2000	>10
Zn_2_**L^4^**	4.8 ± 1.5	>2000	>90
Cu_2_**L^5^**	31.6 ± 2.4	>20,000	>30
Zn_2_**L^5^**	16.9 ± 0.9	>20,000	>100
AMD3100	0.42 ± 0.14	>20,000	>100
Maraviroc	>20,000	2.9 ± 1.2	>100

^a^ Concentration required to inhibit the replication of the HIV-1 strains NL4-3 (X4) and BaL (R5) in TZM-bl cells by 50%. ^b^ Concentration required to reduce cell (PMBCs) viability by 50%. Data are presented as the mean ± SEM of two to five experiments.

**Table 2:** In vitro evaluation of the CXCR4 antagonists in the competition binding and signalling experiments.

Compound	Competition Binding Assay ^a^ IC_50_ (nM)			Ca^2+^ Signalling Assay ^b^ IC_50_ (nM)	
	12G5	CXCL12	CCL5	CXCL12	CCL3L1
Cu_2_**L^1^**	50.1 ± 7.2	5.1 ± 0.9	>1000	113 ± 12.7	>20,000
Zn_2_**L^1^**	5.9 ± 2.4	0.41 ± 0.03	>1000	4.9 ± 2.9	>2000
Cu_2_**L^2^**	465 ± 155	8.9 ± 1.1	>1000	185 ± 75	>20,000
Zn_2_**L^2^**	1.4 ± 0.4	0.43 ± 0.00	255 ± 58	5.6 ± 1.9	120 ± 28
Cu_2_**L^3^**	1.12 ± 0.15	0.54 ± 0.03	741 ± 145	3.1 ± 0.3 [23]	>1000
Zn_2_**L^3^**	1.9 ± 0.3	0.90 ± 0.23	>1000	17.5 ± 11.7	>5000
Cu_2_**L^4^**	1.3 ± 0.4	0.53 ± 0.03	702 ± 219	11.9 ± 8.3	>10,000
Zn_2_**L^4^**	3.4 ± 0.2	2.1 ± 0.1	>1000	66.1 ± 0.9	>20,000
Cu_2_**L^5^**	1.9 ± 0.6	0.83 ± 0.02	>1000	11.3 ± 4.7	>15,000
Zn_2_**L^5^**	8.6 ± 4.7	0.49 ± 0.01	>1000	10.3 ± 6.6	>20,000
AMD3100	83.7 ± 8.3	18.0 ± 4.2 [37]	>1000	203.5 ± 19.4 [23]	>20,000
Maraviroc	-	>1000	10.3 ± 3.5	>20,000	3.1 ± 0.6

^a^ Concentration required to inhibit the binding of PE-conjugated anti-CXCR4 mAb (12G5) in T-lymphocytes or the binding of AF647-labelled chemokines CXCL12 or CCL5 in PMBCs by 50%. ^b^ Concentration required to inhibit chemokine-induced Ca^2+^ signalling in CXCR4- and CCR5-transfected cells by 50%. Data are presented as the mean ± SEM of two to three experiments.

**Table 3:** Change in Gibbs energy (298 K) for single ring chelator model reactions. Results were obtained from the M06/cc-pVDZ calculations.

Single Ring Chelator Model	ΔG_298_ (kJ/mol) [CusL(H_2_O)]^2+^ + OAc^–^ → [CusL(OAc)]^+^ + H_2_O	ΔG_298_ (kJ/mol) [ZnsL(OAc)]^+^ + H_2_O → [ZnsL(OAc)(H_2_O)]^+^	ΔG_298_ (kJ/mol) [ZnsL(H_2_O)_2_]^2+^ + OAc^–^ → [ZnsL(OAc)(H_2_O)]^+^ + H_2_O
**s** **L^1^**	−822.2	−14.9	−825.8
**s** **L^2^**	−800.4	4.0	−
**s** **L^3^**	−833.5	−25.1	−830.6
**s** **L^4^**	−845.9	−23.5	−840.4
**s** **L^5^**	−866.7	−22.5	−849.1

## Data Availability

All data are contained within the article and in the Supplementary Materials.

## Supplementary Materials

The following supporting information can be downloaded at: https://www.mdpi.com/article/10.3390/pharmaceutics16081000/s1, Figure S1: ORTEP representation of [Cu_2_**L^2^**](ClO_4_)_4_; Figure S2: ORTEP representation of [Cu_2_**L^2^**Cl_2_]Cl_2_; Figure S3: Structures of [Zn**sL**(H_2_O)_2_]^2+^ from the DFT calculations; Figure S4: Inhibition of anti-CXCR4 mAb (clone 12G5) binding by the different experimental compounds; Figure S5: Binding inhibition of the fluorescent labelled chemokines CXCL12^AF647^ and CCL5^AF647^; Figure S6: Correlation plots between the biological assays; Table S1: Crystal data and structure refinement for [Cu_2_**L^2^**](ClO_4_)_4_; Table S2: Bond lengths for [Cu_2_**L^2^**](ClO_4_)_4_; Table S3: Bond angles for [Cu_2_**L^2^**](ClO_4_)_4_; Table S4: Crystal data and structure refinement for [Cu_2_**L^2^**Cl_2_]Cl_2_; Table S5: Bond lengths for [Cu_2_**L^2^**Cl_2_]Cl_2_; Table S6: Bond angles for [Cu_2_**L^2^**Cl_2_]Cl_2_.
