# Supplementary material for: Rigid Macrocycle Metal Complexes as CXCR4 Chemokine Receptor Antagonists: Influence of Ring Size
Source: Pharmaceutics. 2024 Jul 28;16(8):1000. doi: 10.3390/pharmaceutics16081000 (PMC11360128; doi:10.3390/pharmaceutics16081000)
Supplement: Supplementary file 1 [file pharmaceutics-16-01000-s001.zip › pharmaceutics-3062337-supplementary.pdf]

Electronic Supplementary Information

Rigid macrocycle metal complexes as CXCR4 chemokine receptor antagonists: influence of ring size

Isaline Renard[1,2], Thomas D'huys[3], Benjamin P. Burke[1], Trisha Ajoleza[4], Amy N. Cain[4], Neil L. Funwie[4], Abid Khan[1,5], Danny L. Maples[4], Randall D. Maples[4], Dallas L. Matz[4], Graeme McRobbie[1], Robert Ullom[4], Timothy J. Prior[6], Douglas P. Linder[4], Tom Van Loy[3], Timothy J. Hubin[4*], Dominique Schols[3*] and Stephen J. Archibald[1,2*]

Crystal structure determination

Single crystals of $C_{32}H_{58}Cl_4Cu_2N_8O_{16}$ ($[Cu_2\mathbf{L}^2](ClO_4)_4$) and $C_{32}H_{58}Cl_4Cu_2N_8O_2$ ($[Cu_2\mathbf{L}^2Cl_2]Cl_2$) were grown by vapor diffusion of diethyl ether at room temperature into a solution of the metal complex in methanol. For each complex, a suitable crystal was selected and mounted on a glass fiber in perfluoropolyether oil on a Stoe IPDS2 diffractometer. The crystal was kept at 150.15 K during data collection using the Oxford Cryosystems Cryostream Cooler. Using Olex2 [1], the structure was solved with the SHELXT [2] structure solution program using Intrinsic Phasing and refined with the SHELXL [3] refinement package using Least Squares minimization.

$[Cu_2L^2](ClO_4)_4$

Crystal data for $C_{32}H_{58}Cl_4Cu_2N_8O_{16}$ (M =1079.74 g/mol): monoclinic, space group $P2_1/n$ (no. 14), a = 17.257(2) Å, b = 15.7102(16) Å, c = 17.976(2) Å, β = 102.416(9)°, V = 4759.6(9) Å^3, Z = 4, T = 150.15 K, μ(MoKα) = 1.189 mm^{-1}, $Dcalc$ = 1.507 g/cm^3, 17160 reflections measured (5.186° ≤ 2Θ ≤ 43.932°), 5806 unique (R_{int} = 0.0567, R_{sigma} = 0.0595) which were used in all calculations. The final R_1 was 0.1304 (I > 2σ(I)) and wR_2 was 0.3674 (all data).

$[Cu_2L^2Cl_2]Cl_2$

Crystal data for $C_{32}H_{58}Cl_4Cu_2N_8O_2$ (M =855.74 g/mol): monoclinic, space group C2/m (no. 12), a = 17.3289(13) Å, b = 9.1582(6) Å, c = 13.5806(13) Å, β = 106.206(7)°, V = 2069.6(3) Å^3, Z = 2, T = 150.15 K, μ(MoKα) = 1.324 mm^{-1}, $Dcalc$ = 1.373 g/cm^3, 8131 reflections measured (5.592° ≤ 2Θ ≤ 54.204°), 2422 unique (R_{int} = 0.0295, R_{sigma} = 0.0262) which were used in all calculations. The final R_1 was 0.0630 (I > 2σ(I)) and wR_2 was 0.2024 (all data).

[1] Dolomanov, O.V., Bourhis, L.J., Gildea, R.J, Howard, J.A.K. & Puschmann, H. (2009), J. Appl. Cryst. 42, 339-341.

[2] Sheldrick, G.M. (2015). Acta Cryst. A71, 3-8.

[3] Sheldrick, G.M. (2015). Acta Cryst. C71, 3-8.

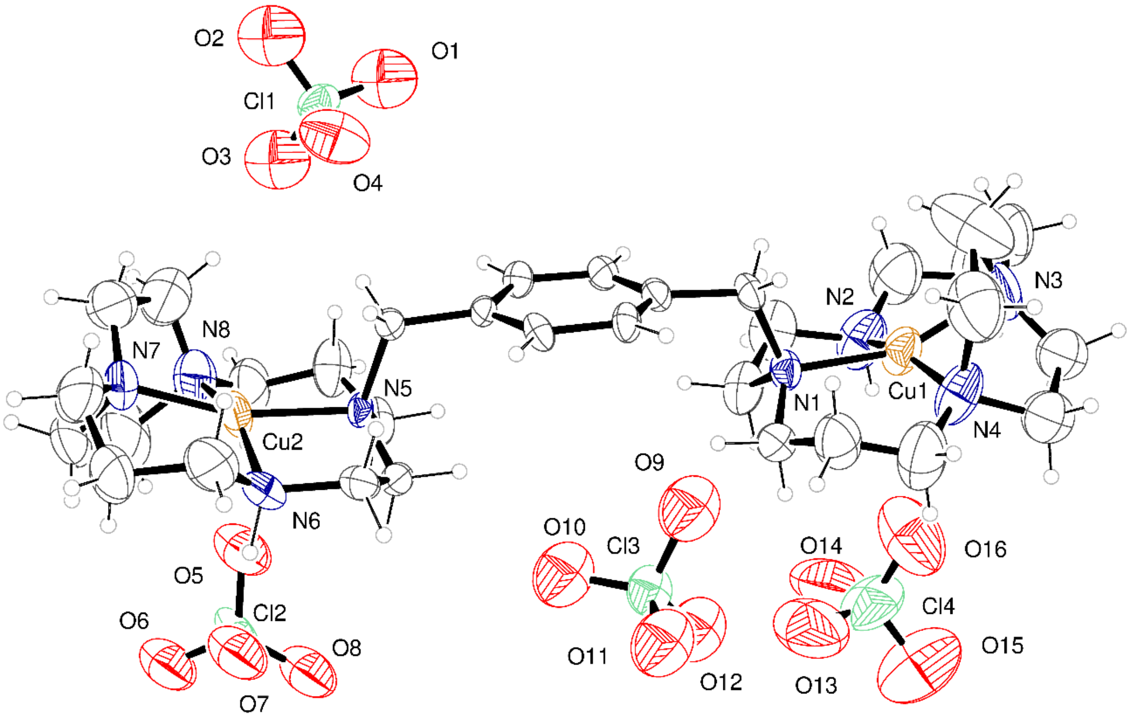

Figure S1. ORTEP representation of $[Cu_2\mathbf{L}^2](ClO_4)_4$ (CCDC number: 2113998) with atoms drawn as 30% probability ellipsoids. Minor disorder in the perchlorate and ethylene bridges is not shown. The whole molecule is contained within the asymmetric unit. Data were rather weak and were trimmed at 0.95 Å.

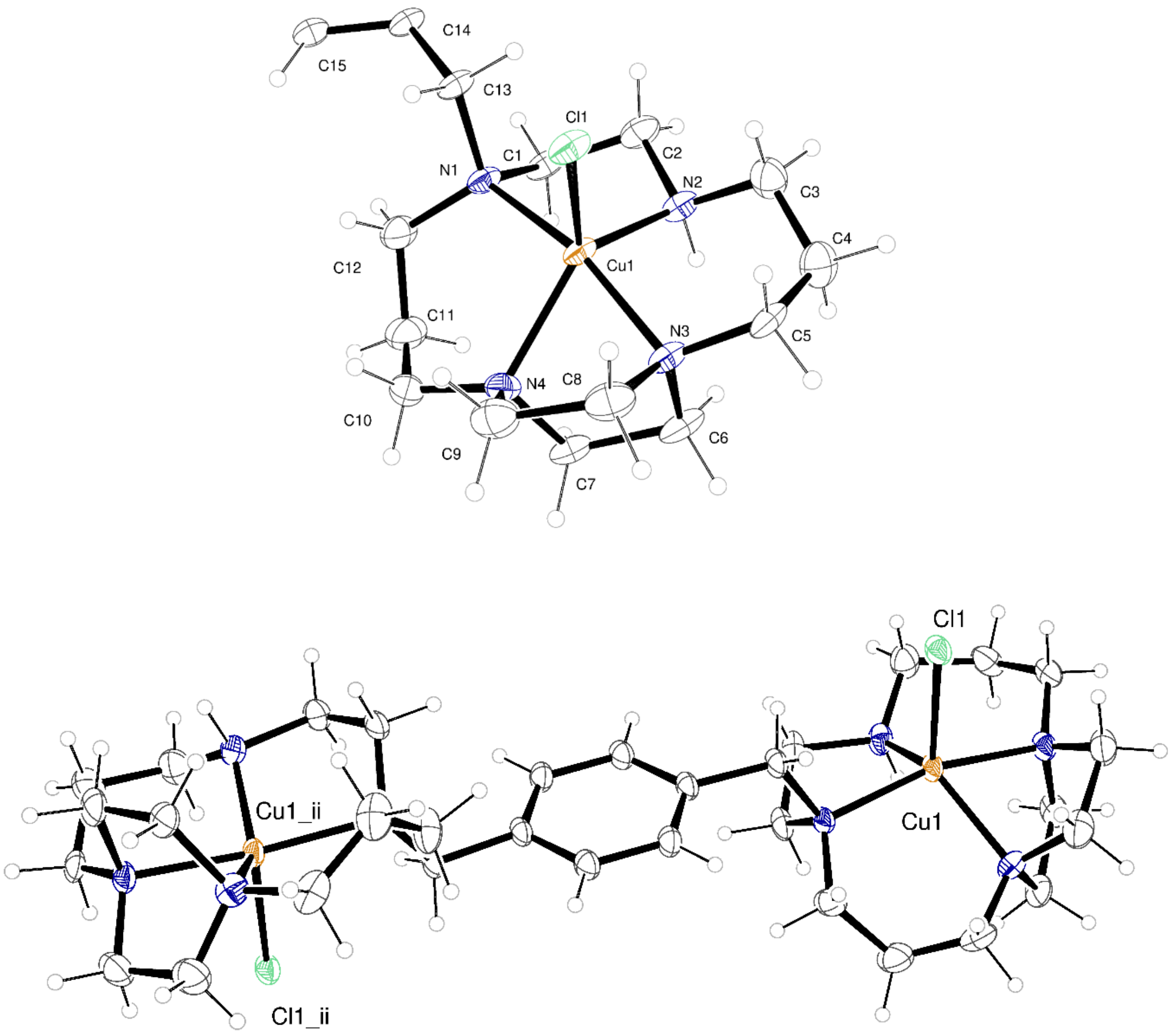

Figure S2. ORTEP representation of [Cu_2**L**^{2}Cl_2]Cl_2 (CCDC number: 2113999) with atoms drawn as 30% probability ellipsoids. Disorder and unbound chloride are not represented. There is one half of the molecule in the asymmetric unit (top). The full molecule is generated by the action of the space group symmetry (bottom). Full molecule present in [Cu_2**L**^{2}Cl_2]Cl_2. Symmetry operator: ii = 2−*x*, *y*, 2−*z*. The analysis of the crystal structure is complicated because the asymmetric unit lies on a crystallographic mirror plane that does not correspond with a mirror plane in the molecule. This generates two orientations for the molecule in equal amounts.

Zn(II) sL1 model

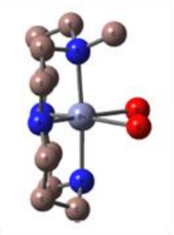

Zn(II) sL2 model

No structure

Zn(II) sL3 model

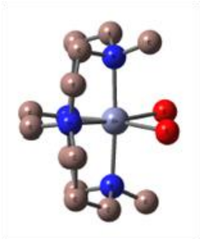

Zn(II) sL4 model

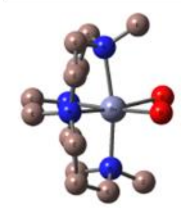

Zn(II) sL5 model

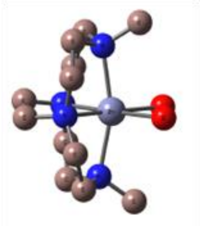

Figure S3. Structures of [Zn**sL**(H_2O)$_2$]$^{2+}$ from DFT calculations.

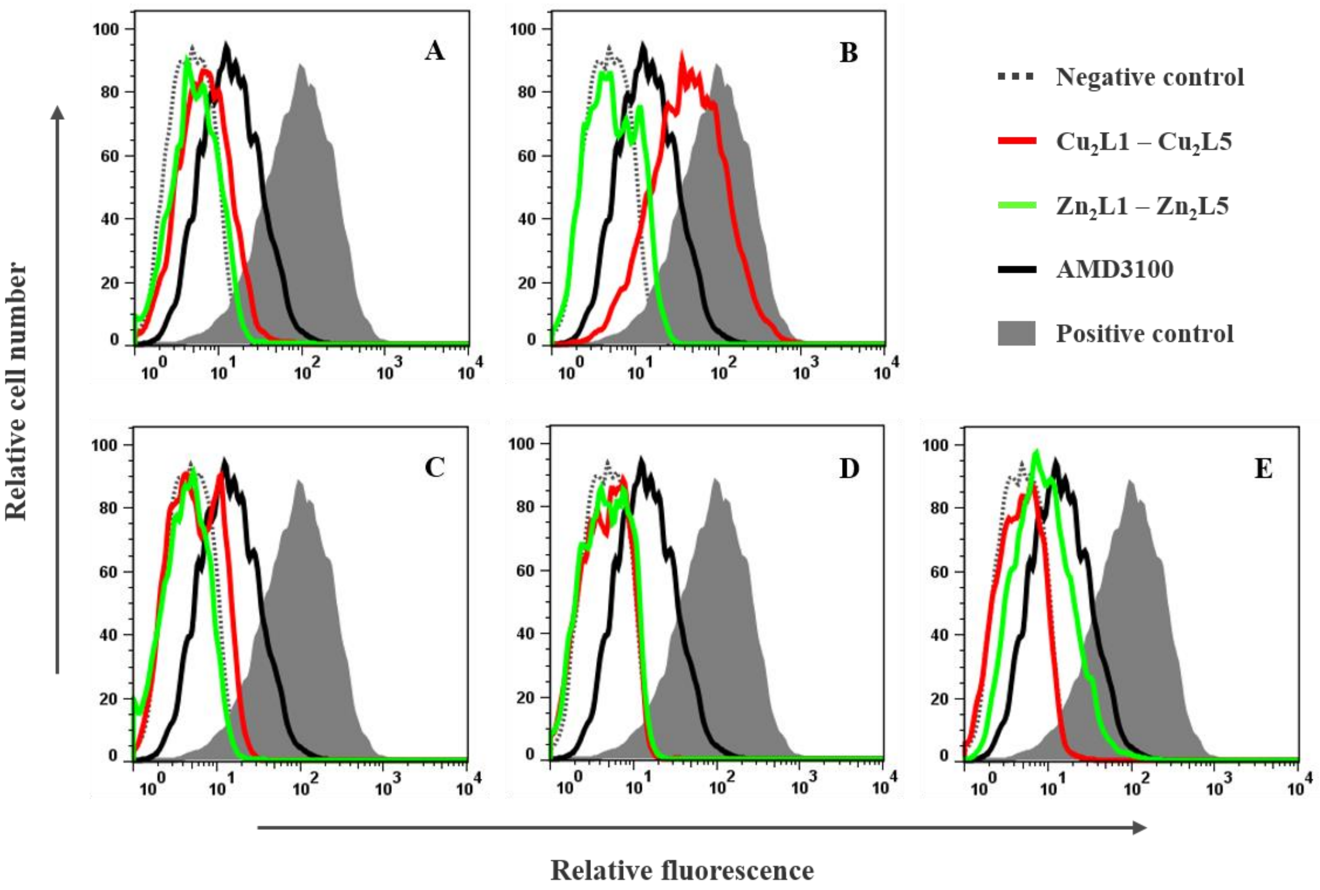

Figure S4. Inhibition of anti-CXCR4 mAb (clone 12G5) binding by the different experimental compounds. Histograms plots were drawn with inhibition profile corresponding to 1 µg/mL of the experimental compounds and the CXCR4 antagonist AMD3100. Each panel of this figure (A to E) shows a combination of the corresponding histogram plots: negative control (dashed line), $Cu_2\mathbf{L^1}$ – $Cu_2\mathbf{L^5}$ (red line), $Zn_2\mathbf{L^1}$ – $Zn_2\mathbf{L^5}$ (green line), AMD3100 (black line) and positive control (grey).

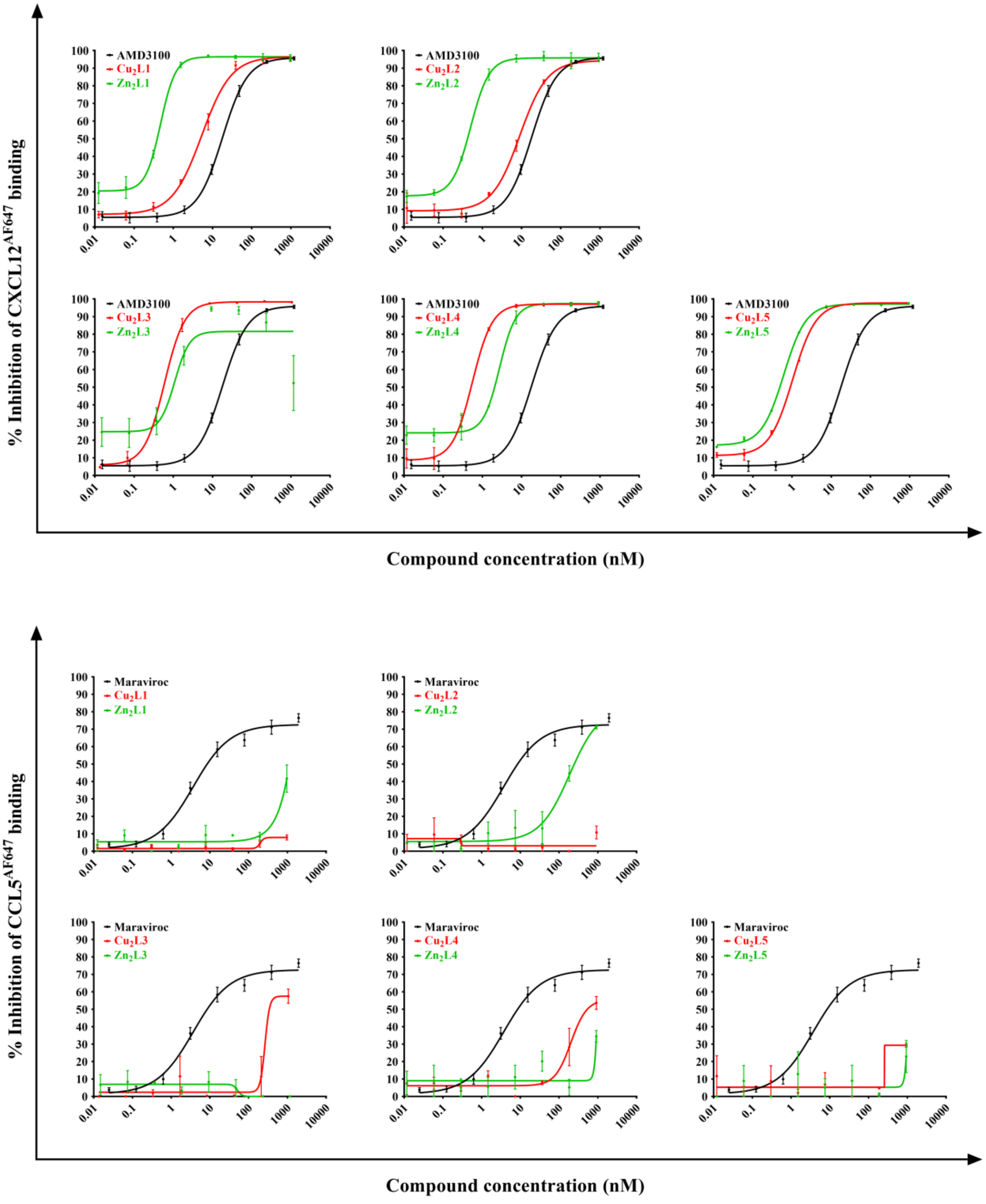

Figure S5. Binding inhibition of the fluorescent labeled chemokines $CXCL12^{AF647}$ (top) and $CCL5^{AF647}$ (bottom) in PBMCs after pre-incubation with the serial dilutions of the experimental compounds.

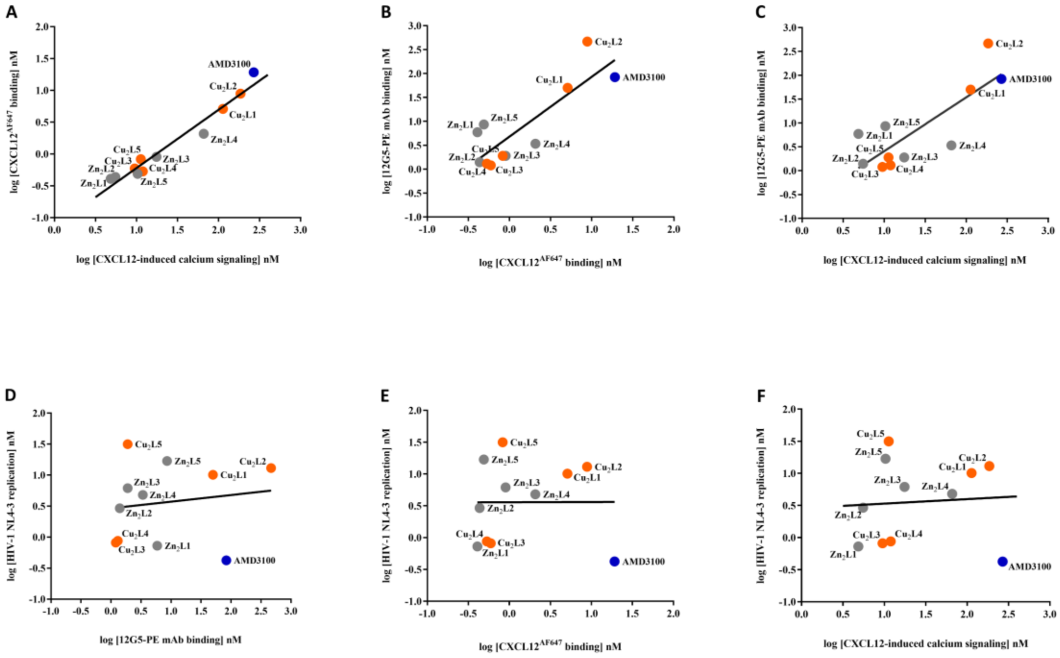

Figure S6. Correlation plots between the biological assays.

Table S1. Crystal data and structure refinement for $[Cu_2\mathbf{L}^2](ClO_4)_4$.

Identification code	sja8_04
Empirical formula	$C_{32}H_{58}Cl_4Cu_2N_8O_{16}$
Formula weight	1079.74
Temperature/K	150.15
Crystal system	monoclinic
Space group	$P2_1/n$
a/Å	17.257(2)
b/Å	15.7102(16)
c/Å	17.976(2)
α/°	90
β/°	102.416(9)
γ/°	90
Volume/Å^3	4759.6(9)
Z	4
ρ_{calc}g/cm^3	1.507
μ/mm^{-1}	1.189
F(000)	2240.0
Crystal size/mm^3	0.2 × 0.1 × 0.1
Radiation	MoKα (λ = 0.71073)
2Θ range for data collection/°	5.186 to 43.932
Index ranges	$-18 \le h \le 18$, $-16 \le k \le 16$, $-18 \le l \le 17$
Reflections collected	17160
Independent reflections	5806 [R_{int} = 0.0567, R_{sigma} = 0.0595]
Data/restraints/parameters	5806/997/552
Goodness-of-fit on F^2	1.305
Final R indexes [I>=2σ (I)]	R_1 = 0.1304, wR_2 = 0.3387
Final R indexes [all data]	R_1 = 0.1680, wR_2 = 0.3674
Largest diff. peak/hole / e Å^{-3}	1.41/-0.64

Table S2. Bond lengths for $[Cu_2\mathbf{L^2}](ClO_4)_4$.

Atom	Atom	Length/Å	Atom	Atom	Length/Å
Cu1	N1	1.990(8)	C11	C12	1.67(4)
Cu1	N2	2.073(17)	C13	C14	1.70(3)
Cu1	N3	1.893(14)	C15	C16	1.68(4)
Cu1	N4	1.879(14)	C17	C18	1.34(2)
Cu2	N5	1.980(7)	C18	C19	1.49(2)
Cu2	N6	2.237(15)	C21	C22	1.520(19)
Cu2	N7	1.933(11)	C23	C24	1.56(3)
Cu2	N8	1.871(16)	C24	C25	1.48(3)
N1	C7	1.521(14)	C26	C27	1.52(3)
N1	C8	1.502(12)	C28	C29	1.78(4)
N1	C19	1.493(12)	C30	C31	1.45(2)
N2	C9	1.528(14)	C31	C32	1.47(2)
N2	C10	1.484(15)	Cl2	O5	1.390(16)
N3	C12	1.480(15)	Cl2	O6	1.366(17)
N3	C13	1.464(16)	Cl2	O7	1.396(18)
N3	C15	1.64(4)	Cl2	O8	1.382(17)
N3	C13A	1.499(16)	Cl1	O1	1.370(16)
N4	C14	1.511(15)	Cl1	O2	1.375(18)
N4	C16	1.530(15)	Cl1	O3	1.365(18)
N4	C17	1.461(14)	Cl1	O4	1.375(15)
N4	C14A	1.501(16)	O4	Cl1A	1.392(16)
N5	C20	1.512(12)	Cl3	O9	1.400(16)
N5	C21	1.466(11)	Cl3	O10	1.352(16)
N5	C32	1.464(11)	Cl3	O11	1.380(17)
N6	C22	1.474(12)	Cl3	O12	1.389(16)
N6	C23	1.438(14)	Cl4	O13	1.380(15)
N7	C25	1.461(14)	Cl4	O14	1.386(17)
N7	C27	1.412(14)	Cl4	O15	1.339(15)
N7	C29	1.434(13)	Cl4	O16	1.351(15)
N8	C26	1.444(14)	Cl1A	O1A	1.383(18)
N8	C28	1.452(14)	Cl1A	O2A	1.359(19)
N8	C30	1.400(14)	Cl1A	O3A	1.380(19)
C1	C2	1.386(15)	Cl3A	O9A	1.408(18)
C1	C6	1.347(15)	Cl3A	O11A	1.374(19)
C1	C7	1.521(14)	Cl3A	O10A	1.356(18)
C2	C3	1.390(13)	Cl3A	O12A	1.44(4)
C3	C4	1.403(14)	O7A	Cl2A	1.377(18)
C4	C5	1.385(15)	Cl2A	O8A	1.395(17)
C4	C20	1.503(13)	Cl2A	O6A	1.356(18)
C5	C6	1.389(14)	Cl2A	O5A	1.400(17)
C8	C9	1.50(2)	C13A	C14A	1.72(3)
C10	C11	1.41(4)			

Table S3. Bond angles for $[Cu_2\mathbf{L^2}](ClO_4)_4$.

Atom	Atom	Atom	Angle/°	Atom	Atom	Atom	Angle/°
N1	Cu1	N2	88.5(5)	C1	C7	N1	115.3(8)
N3	Cu1	N1	154.7(7)	C9	C8	N1	107.8(13)
N3	Cu1	N2	98.5(7)	C8	C9	N2	108.8(14)
N4	Cu1	N1	99.6(5)	C11	C10	N2	111(2)
N4	Cu1	N2	160.6(6)	C10	C11	C12	106(3)
N4	Cu1	N3	81.7(7)	N3	C12	C11	111(2)
N5	Cu2	N6	84.2(4)	N3	C13	C14	106.1(17)
N7	Cu2	N5	160.5(4)	N4	C14	C13	102.0(17)
N7	Cu2	N6	94.0(6)	N3	C15	C16	90(2)
N8	Cu2	N5	106.1(5)	N4	C16	C15	115(2)
N8	Cu2	N6	164.2(5)	C18	C17	N4	111.5(17)
N8	Cu2	N7	80.2(7)	C17	C18	C19	123(2)
C7	N1	Cu1	109.8(6)	C18	C19	N1	113.2(12)
C8	N1	Cu1	105.2(7)	C4	C20	N5	115.2(7)
C8	N1	C7	110.3(9)	N5	C21	C22	111.2(9)
C19	N1	Cu1	111.9(7)	N6	C22	C21	108.4(11)
C19	N1	C7	109.6(8)	N6	C23	C24	106.2(18)
C19	N1	C8	110.0(10)	C25	C24	C23	112.5(17)
C9	N2	Cu1	101.7(10)	N7	C25	C24	112.0(17)
C10	N2	Cu1	115.0(16)	N8	C26	C27	104.7(18)
C10	N2	C9	111.8(17)	N7	C27	C26	112.7(17)
C12	N3	Cu1	121.6(14)	N8	C28	C29	106.5(17)
C12	N3	C15	103(2)	N7	C29	C28	100.2(13)
C12	N3	C13A	115.3(19)	N8	C30	C31	116.3(16)
C13	N3	Cu1	101.6(18)	C30	C31	C32	116.8(17)
C13	N3	C12	109.0(18)	N5	C32	C31	112.5(10)
C13	N3	C15	132(3)	O5	Cl2	O7	102.2(15)
C15	N3	Cu1	90.7(14)	O6	Cl2	O5	112.9(18)
C13A	N3	Cu1	110.6(16)	O6	Cl2	O7	110.5(19)
C13A	N3	C15	112(3)	O6	Cl2	O8	113.7(17)
C14	N4	Cu1	109.9(15)	O8	Cl2	O5	113.2(17)
C14	N4	C16	99.8(14)	O8	Cl2	O7	103.2(17)
C16	N4	Cu1	94.8(14)	O1	Cl1	O2	108.6(16)
C17	N4	Cu1	123.9(13)	O1	Cl1	O4	115.6(16)
C17	N4	C14	111.7(15)	O2	Cl1	O4	104.1(14)
C17	N4	C16	113.0(18)	O3	Cl1	O1	111.4(16)
C17	N4	C14A	112.5(19)	O3	Cl1	O2	111.5(17)
C14A	N4	Cu1	93(2)	O3	Cl1	O4	105.5(14)
C14A	N4	C16	118.1(19)	O10	Cl3	O9	107.5(15)
C20	N5	Cu2	107.6(5)	O10	Cl3	O11	112.9(15)
C21	N5	Cu2	107.5(6)	O10	Cl3	O12	115.4(15)
C21	N5	C20	112.3(8)	O11	Cl3	O9	103.5(14)
C32	N5	Cu2	108.8(6)	O11	Cl3	O12	107.5(14)

Atom	Atom	Atom	Angle/°	Atom	Atom	Atom	Angle/°
C32	N5	C20	110.3(9)	O12	Cl3	O9	109.3(16)
C32	N5	C21	110.1(9)	O13	Cl4	O14	101.0(15)
C22	N6	Cu2	104.0(9)	O15	Cl4	O13	108.2(14)
C23	N6	Cu2	113.5(12)	O15	Cl4	O14	108.7(14)
C23	N6	C22	115.9(14)	O15	Cl4	O16	115.0(16)
C25	N7	Cu2	126.3(12)	O16	Cl4	O13	115.0(12)
C27	N7	Cu2	102.3(11)	O16	Cl4	O14	107.9(14)
C27	N7	C25	115.4(16)	O1A	Cl1A	O4	114.8(19)
C27	N7	C29	105.5(18)	O2A	Cl1A	O4	113.0(17)
C29	N7	Cu2	103.6(10)	O2A	Cl1A	O1A	107.7(19)
C29	N7	C25	101.4(16)	O2A	Cl1A	O3A	107(2)
C26	N8	Cu2	102.1(15)	O3A	Cl1A	O4	106.8(18)
C26	N8	C28	109.5(19)	O3A	Cl1A	O1A	106.7(19)
C28	N8	Cu2	101.7(15)	O9A	Cl3A	O12A	96(3)
C30	N8	Cu2	114.7(13)	O11A	Cl3A	O9A	104(2)
C30	N8	C26	118.0(19)	O11A	Cl3A	O12A	105(2)
C30	N8	C28	109.5(18)	O10A	Cl3A	O9A	106(2)
C2	C1	C7	118.2(10)	O10A	Cl3A	O11A	112(2)
C6	C1	C2	119.8(9)	O10A	Cl3A	O12A	130(3)
C6	C1	C7	121.9(10)	O7A	Cl2A	O8A	102.7(18)
C1	C2	C3	119.7(10)	O7A	Cl2A	O5A	108.3(18)
C2	C3	C4	120.9(10)	O8A	Cl2A	O5A	113(2)
C3	C4	C20	121.8(9)	O6A	Cl2A	O7A	108(2)
C5	C4	C3	117.4(8)	O6A	Cl2A	O8A	118.3(19)
C5	C4	C20	120.9(9)	O6A	Cl2A	O5A	106.0(18)
C4	C5	C6	120.9(10)	N3	C13A	C14A	100(2)
C1	C6	C5	121.1(10)	N4	C14A	C13A	98(2)

Table S4. Crystal data and structure refinement for $[Cu_2\mathbf{L}^2Cl_2]Cl_2$.

Identification code	sja8_02
Empirical formula	$C_{32}H_{58}Cl_4Cu_2N_8O_2$
Formula weight	855.74
Temperature/K	150.15
Crystal system	monoclinic
Space group	C2/m
a/Å	17.3289(13)
b/Å	9.1582(6)
c/Å	13.5806(13)
α/°	90
β/°	106.206(7)
γ/°	90
Volume/Å^3	2069.6(3)
Z	2
ρ_{calc}g/cm^3	1.373
μ/mm^{-1}	1.324
F(000)	896.0
Crystal size/mm^3	0.2 × 0.2 × 0.15
Radiation	MoKα (λ = 0.71073)
2Θ range for data collection/°	5.592 to 54.204
Index ranges	$-18 \leq h \leq 22$, $-11 \leq k \leq 11$, $-17 \leq l \leq 17$
Reflections collected	8131
Independent reflections	2422 [R_{int} = 0.0295, R_{sigma} = 0.0262]
Data/restraints/parameters	2422/203/194
Goodness-of-fit on F^2	1.066
Final R indexes [I>=2σ (I)]	R_1 = 0.0630, wR_2 = 0.1934
Final R indexes [all data]	R_1 = 0.0714, wR_2 = 0.2024
Largest diff. peak/hole / e Å^{-3}	2.03/-0.84

Table S5. Bond lengths for $[Cu_2\mathbf{L^2}Cl_2]Cl_2$.

Atom	Atom	Length/Å	Atom	Atom	Length/Å
Cu1	Cl1	2.4133(14)	C14	C13	1.514(9)
Cu1	N3	2.040(4)	C6	C7	1.650(10)
Cu1	N1	2.094(5)	C12	C11	1.482(13)
Cu1	N2	1.938(7)	N2	C2	1.513(10)
Cu1	N4	2.141(6)	N2	C3	1.489(12)
N3	C6	1.490(7)	C9	N4	1.422(15)
N3	C8	1.50(4)	C9	C8	1.63(4)
N3	C5	1.46(4)	C7	N4	1.506(10)
N1	C13	1.511(12)	C2	C1	1.521(14)
N1	C12	1.498(11)	N4	C10	1.490(11)
N1	C1	1.504(11)	C10	C11	1.466(15)
C15	C15[1]	1.386(7)	C3	C4	1.485(15)
C15	C14	1.396(5)	C4	C5	1.48(4)

[1]2-X,+Y,2-Z

Table S6. Bond angles for $[Cu_2\mathbf{L}^2Cl_2]Cl_2$.

Atom	Atom	Atom	Angle/°	Atom	Atom	Atom	Angle/°
N3	Cu1	Cl1	92.56(13)	C15	C14	C13	117(3)
N3	Cu1	N1	165.51(19)	$C15^2$	C14	C13	125(3)
N3	Cu1	N4	70.3(2)	N3	C6	C7	101.9(4)
N1	Cu1	Cl1	99.69(15)	N1	C13	C14	116.2(6)
N1	Cu1	N4	97.3(3)	C11	C12	N1	118.1(9)
N2	Cu1	Cl1	110.0(2)	C2	N2	Cu1	108.3(5)
N2	Cu1	N3	94.0(2)	C3	N2	Cu1	111.3(5)
N2	Cu1	N1	89.1(3)	C3	N2	C2	114.5(7)
N2	Cu1	N4	133.2(3)	N4	C9	C8	106.7(17)
N4	Cu1	Cl1	114.45(18)	N4	C7	C6	107.3(6)
C6	N3	Cu1	103.5(3)	N2	C2	C1	105.2(7)
C6	N3	C8	115.3(14)	C9	N4	Cu1	105.0(7)
C8	N3	Cu1	106.5(15)	C9	N4	C7	111.2(8)
C5	N3	Cu1	113.3(13)	C9	N4	C10	110.0(9)
C5	N3	C6	106.9(13)	C7	N4	Cu1	101.7(5)
C5	N3	C8	111.3(6)	C10	N4	Cu1	118.7(5)
C13	N1	Cu1	110.2(7)	C10	N4	C7	109.9(7)
C12	N1	Cu1	115.8(5)	C11	C10	N4	112.4(7)
C12	N1	C13	109(3)	C4	C3	N2	110.9(9)
C12	N1	C1	113.0(8)	C10	C11	C12	113.3(9)
C1	N1	Cu1	99.9(4)	N1	C1	C2	111.7(7)
C1	N1	C13	109(3)	C5	C4	C3	117.6(18)
$C15^1$	C15	C14	121.0(2)	N3	C8	C9	104(2)
C15	C14	$C15^2$	117.9(4)	N3	C5	C4	116(2)

[1]2-X,+Y,2-Z; [2]+X,1-Y,+Z
